# Supplementary material for: Quantifying gender biases towards politicians on Reddit
Source: PLoS One. 2022 Oct 26;17(10):e0274317. doi: 10.1371/journal.pone.0274317 (PMC9603992; doi:10.1371/journal.pone.0274317)
Supplement: S1 Table — (PDF) [file pone.0274317.s001.pdf]

S1 Table. Subreddits Included.

**Subreddit**	**Number of comments**	**Partisan-affiliation**
politics	9744853	—
The_Donald	1664335	alt-right
news	556783	—
neoliberal	340533	left
canada	285667	—
Libertarian	207109	right
Conservative	200772	right
unitedkingdom	197881	—
europe	158342	—
australia	107966	—
india	87367	—
democrats	53381	left
ireland	40964	—
teenagers	33311	—
newzealand	32847	—
socialism	18241	left
TwoXChromosomes	15734	—
MensRights	13664	—
Republican	13014	right
Liberal	10503	left
uspolitics	8873	—
SocialDemocracy	1977	left
alltheleft	837	left
feminisms	108	—
