# Supplementary material for: Quantifying gender biases towards politicians on Reddit
Source: PLoS One. 2022 Oct 26;17(10):e0274317. doi: 10.1371/journal.pone.0274317 (PMC9603992; doi:10.1371/journal.pone.0274317)
Supplement: S2 Table — (PDF) [file pone.0274317.s002.pdf]

S2 Table. PMI Annotations.

Female-bias words	Sense	Sentiment	Male-bias words	Senses	Sentiment
chairwoman	Profession	0	bloke	Label	0
pantsuit	Clothing	0	wanker	Label	-1
matriarch	Family	0	prince	Profession	0
facelift	Body	0	lawful	Belief	1
menopausal	Attribute	-1	turkish	Other	0
harpy	Label	-1	madman	Attribute	-1
scarf	Clothing	0	unchecked	Attribute	-1
clitoris	Body	0	punchable	Body	-1
clit	Body	-1	dickhead	Label	-1
brunette	Body	0	truck	Other	0
wench	Label	-1	businessman	Profession	0
hind	Body	-1	informant	Other	0
childless	Family	0	inflation	Other	-1
skank	Label	-1	jock	Label	-1
misandrist	Attribute	-1	sovereign	Other	1
hubby	Family	0	chode	Label	-1
cheekbone	Body	0	prick	Label	-1
boogeywoman	Label	-1	cure	Other	1
btch	Label	-1	envoy	Profession	0
brooch	Clothing	0	testicle	Body	0
nosedive	Other	-1	ruler	Profession	0
driven	Attribute	0	worm	Other	-1
conceal	Other	-1	republic	Belief	0
elegance	Attribute	0	discovery	Other	1
ballz	Label	-1	douchebag	Label	-1
numerical	Other	0	constitutionalist	Belief	0
goddess	Body	1	urgent	Other	0
blouse	Clothing	0	lad	Label	-1
interjection	Other	0	hereby	Other	0
succubus	Label	-1	mafia	Other	-1
heroine	Attribute	1	bluster	Attribute	-1
aunty	Family	0	imperialism	Belief	-1
equipped	Attribute	0	kiddie	Label	-1
progressiveness	Belief	0	undisclosed	Other	-1
dependency	Other	0	kisser	Attribute	-1
congresswoman	Profession	0	sleazeball	Label	-1

**Female-bias words**	**Sense**	**Sentiment**	**Male-bias words**	**Senses**	**Sentiment**
hag	Label	-1	errand	Other	0
fuckable	Body	-1	gatekeeper	Attribute	0
frumpy	Body	-1	chairman	Profession	0
racy	Clothing	-1	longstanding	Other	0
ovary	Body	0	shitbird	Label	-1
smokey	Other	0	douche	Label	-1
crone	Body	-1	fanboy	Label	0
dyke	Label	-1	congressman	Profession	0
suffragette	Belief	1	excess	Other	0
businesswoman	Profession	0	diddler	Label	-1
42nd	Other	-1	manifestation	Other	0
radiant	Attribute	1	utopia	Belief	1
charmed	Attribute	0	federalist	Belief	0
mediator	Attribute	0	meddling	Attribute	-1
throatedly	Attribute	-1	lowlife	Label	-1
regal	Attribute	0	alley	Other	0
skincare	Body	0	ukraine	Other	0
biatch	Label	-1	chancellor	Profession	0
wonkiness	Other	-1	affect	Other	-1
finely	Attribute	0	offshore	Other	-1
statesperson	Attribute	0	philosophical	Attribute	0
memelord	Label	-1	grim	Attribute	-1
stepmother	Family	0	wimp	Label	-1
peopel	Other	0	rain	Other	0
dementor	Label	-1	crypto	Other	0
cackle	Attribute	-1	henchman	Profession	-1
shrew	Label	-1	overhaul	Other	0
mudslinging	Attribute	-1	palace	Other	0
skeletor	Body	-1	nationalistic	Belief	-1
jewelry	Clothing	0	erection	Body	0
stepmom	Family	0	domain	Other	0
monstrously	Other	-1	sleaze	Label	-1
homewrecker	Label	-1	pronouncement	Other	0
palestine	Other	0	locker	Other	0
bossy	Attribute	-1	clique	Other	-1
tremor	Body	-1	clickbait	Other	-1
stepford	Label	-1	plausibly	Other	0
bangable	Body	-1	subway	Other	0
fugly	Label	-1	fella	Label	0
supermodel	Body	-1	slimeball	Label	-1
antivax	Belief	-1	fuckhead	Label	-1
campaign-	Other	0	secular	Belief	0
poised	Attribute	0	criminality	Attribute	-1
headscarf	Clothing	-1	goof	Label	-1

**Female-bias words**	**Sense**	**Sentiment**	**Male-bias words**	**Senses**	**Sentiment**
spokeswoman	Profession	0	christ	Other	0
horseface	Body	-1	horde	Other	-1
hottie	Body	-1	usd	Other	0
sow	Label	-1	onwards	Other	0
ditzy	Attribute	-1	biblical	Attribute	0
yesrep	Other	-1	expendable	Attribute	0
usurping	Profession	-1	stunned	Attribute	0
gmo	Other	0	founder	Profession	0
inescapable	Attribute	-1	behest	Other	0
flashlight	Other	0	monarch	Profession	0
qualify	Other	0	priest	Profession	1
parkinson	Body	-1	jazz	Other	0
detectable	Other	0	mogul	Profession	-1
pizzeria	Other	0	obedient	Attribute	-1
grannie	Family	0	soy	Other	0
hom	Other	0	ping	Other	0
authortarian	Belief	-1	metal	Other	0
fairweather	Attribute	0	asswipe	Label	-1
peen	Body	-1	passage	Other	0
sourpuss	Attribute	-1	wingnut	Label	-1
