# Supplementary material for: Quantifying gender biases towards politicians on Reddit
Source: PLoS One. 2022 Oct 26;17(10):e0274317. doi: 10.1371/journal.pone.0274317 (PMC9603992; doi:10.1371/journal.pone.0274317)
Supplement: S1 Text — (PDF) [file pone.0274317.s003.pdf]

S3 Text. Removal of Trump.

One reviewer suggested, given the overwhelming prevalence of comments discussing Donald Trump in our data-set, that we double-check all our findings still hold with the removal of all Trump-related comments from our data-set. Here are the updated results for all analyses that did not already take into consideration skews in politician popularity (i.e. analyses that relied on parametric statistical tests, such as Student t-tests and chi-square tests). This updated data-set (with all comments determined to mention Donald Trump removed) now consists of 5,951,271 comments. 4,957,699 comments only mention a single politician (and are used for the majority of the following analyses). 1,057,017 of these comments are included in the partisan data-set. Given that Donald Trump is a man, we only report results that relate to men (as analyses that only look into woman-containing comments are unlikely to have changed).

Coverage biases

We see slightly longer average comments after the removal of Trump from the dataset. While the average length of comments discussing male politicians (43.33 ± 62.11 tokens) is significantly longer than comments discussing female politicians ($t(4957698) = 78.10, p < .0001$), the effect size of the difference also remains negligible (Cohen's D: 0.08).

When it comes to the cross-partisan comparison, we again see similar results. There is a significant main effect of sex ($F(1, 1056932) = 2638.8, p < .0001$) and partisanship ($F(2, 1056932) = 9786.7, p < .0001$) as well as a significant interaction ($F(2, 1056932) = 410.9, p < .0001$). Post-hoc Tukey HSD tests show that comments about men ($\mu = 38.0 \pm 60.6$ tokens), while shorter than previously reported, are still significantly longer than comments about women ($p < .0001; d = 0.12$), but the effect size is still negligible. Comments on right-leaning subreddits ($\mu = 53.3 \pm 82.3$) are significantly longer than those on the left ($\mu = 42.2 \pm 72.9, p < .0001, d = 0.14$) and alt-right ($\mu = 31.5 \pm 45.0, p < .0001, d = 0.41$). Left-leaning comments remain longer than those on the alt-right ($p < .0001, d = 0.21$). All interaction differences were significant, though negligible and, therefore, not reported ($d < 0.2$). Therefore, we see similar results as reported with Trump-containing comments.

Combinatorial Biases

Re-calculating $L(g_{given}, g_{add})$ with all Trump-containing comments removed, we are left with 993,572 unique comments discussing 2,401,577 individuals. The new $L(g_{given}, g_{add})$ values are reported in Table S1. We see a similar pattern as seen in the Trump-containing datasets. Looking at values that share a g_{add}, we can still see heterophily, though it seems that we see slightly more homophily for female politicians than in the Trump-removed data-sets (as there is a smaller gap between the two L values). In addition, our null model permutations suggest that the observed values have a $p < 10^{-5}$.

		g_{given}	
		**female**	**male**
g_{add}	**female**	0.20	0.21
	**male**	1.16	0.97

Table 1. Recorded values of $L(g_{given}, g_{add})$.

When it comes to the cross-partisanal analyses, we again find that all observed values have a p-value of under 10^{-5}. The observed L values are reported in Table S2.

		Left		Right		Alt-right	
		g_{given}		g_{given}		g_{given}	
		**male**	**female**	**male**	**female**	**male**	**female**
g_{add}	**male**	1.07	1.28	1.04	1.11	0.90	1.02
	**female**	0.20	0.21	0.18	0.23	0.24	0.23

Table 2. Recorded values of $L(g_{given}, g_{add})$ on the cross-partisan dataset

Once more we can see that men are more likely to appear in the context in women. Again we see, in the left- and right-leaning data splits, women are more likely to be observed in the context in other women, than men. This is flipped in the alt-right subreddits. Therefore, though the observed L is different with the removal of Trump, we continue to see a similar pattern of slight homophily between female politicians in left- and right-leaning subreddits, which is not observed in the alt-right subreddit.

Nominal biases

We continue to see a similar pattern in how politicians are named, though the specific values achieved via odds ratios have changed. A chi-square test of independence still finds a significant relation between subject gender and reference used $\chi^2(3, N = 4957165) = 682401, p < .0001, V = .37)$. While male politicians are now only referred by their surname in 53.0% of all instances (relative to 69.7%), odds ratios still show the odds of a male politician being named by his surname is 4.43 times greater than for a female politician $(95\% CI : 4.41 - 4.45, p < .0001)$. The odds of a female politician being named by her first name are 7.62 times greater than for a male politician $(95\% CI : 7.57 - 7.68, p < .0001)$. We also see the female politicians have 2.62 times greater odds than men of being named by their full name $(95\% CI : 2.61 - 2.63, p < .0001)$

When we look across partisan divides, a three-way log-linear analysis produces a final model retaining all effects with likelihood ratio of $\chi^2(0) = 0, p = 1)$, indicating again that the highest-order interaction is significant. Further separate chi-square tests on the two-way interactions find that there is a significant association between politician gender and choice of nomination in left-leaning $\chi^2(3) = 21559, p < .0001, V = .34)$, right-leaning $\chi^2(3) = 25311, p < .0001, V = .41)$, and alt-right subreddits $\chi^2(3) = 142641, p < .0001, V = .44)$. We still find a significant association between partisanship and choice of nomination for male politicians $\chi^2(6) = 1444.8, p < .0001, V = .03)$. This matches what we observed in the Trump-containing data-set. In alt-right subreddits, the odds-ratio for a women to be named by her given name is 10.4 times greater than for men $(95\% CI : 10.24 - 10.58, p < .0001)$. In right-leaning subreddits, the odds are 7.75 times greater for a women than a man to be named by a given name $(95\% CI : 7.44 - 8.07, p < .0001)$. Likewise, in left-leaning subreddits, the odds for a woman to be named by her given name is 5.66 times greater than a man $(95\% CI : 5.47 - 5.85, p < .0001)$. Though the odds are smaller than seen when Trump is included in the data-set, we see a similar pattern as before (and, in alt-right subreddits, the odds for women to be named by her given name relative to a man is again nearly double that seen in left-leaning subreddits). Men are now only 4.76 times more likely to be named by their surname than women in right-leaning subreddits $(95\% CI : 4.61 - 4.92, p < .0001)$. Similary, in alt-right leaning subreddits, men have 4.63 greater odds of being named by their surname than women $(95\% CI : 4.57 - 4.68, p < .0001)$. In left-leaning subreddits, though the odds are still

smaller than seen in the right and alt-right leaning subreddits, the odds for a man to be named by their surname is still 3.63 greater than a woman ($95\% CI: 3.53 - 3.73, p < .0001$). Overall, we see a very similar pattern of results as in the Trump-containing dataset.

Sentimental biases

We see similar average lexicon-based valence and dominance values as before the removal of Trump. The average valence of comments discussing male politicians (0.324 ± 0.205) are still significantly more positive than comments discussing female politicians ($t(4957698) = 42.60, p < .0001$). However, the effect size of the difference remains negligible ($d: 0.05$). The average dominance of comments discussing male politicians (0.298 ± 0.187) is still significantly greater than comments discussing female politicians ($t(4957698) = 62.27, p < .0001$). However, the effect size of the difference remains negligible (Cohen's D: 0.06).

When it comes to the classifier-based method, a chi-square test of independence still finds a significant (though negligible) relation between subject gender and output sentiment rating $\chi^2(1, N = 4957165) = 204.51, p < .0001, V = .01$)

In the cross-partisan comparison, we see similar results.

Following the lexicon-based method, we find a significant main effect of sex ($F(1, 1056932) = 735.1, p < .0001$) and partisanship ($F(2, 1056932) = 3464.7, p < .0001$) on comment Valence as well as a significant interaction ($F(2, 1056932) = 271.1, p < .0001$). Post-hoc Tukey HSD tests show that comments about men ($\mu = 0.337 \pm 0.10$) are still more positive than comments about women ($p < .0001; d = 0.06$), but the effect size is still negligible. Comments on alt-right communities ($\mu = 0.344 \pm 0.211$) are significantly more positive than those on the left ($\mu = 0.301 \pm 0.200, p < .0001, d = 0.21$) and right ($\mu = 0.323 \pm 0.205, p < .0001, d = 0.10$). Right-leaning comments remain more positive than those on the left ($p < .0001, d = 0.11$). While many interaction differences were significant, all differences in comment valence were negligible ($d < 0.2$), similarly to what was reported in the Trump-containing dataset.

We find a significant main effect of sex ($F(1, 1056932) = 1431.8, p < .0001$) and partisanship ($F(2, 1056932) = 2733.5, p < .0001$) on comment Dominance as well as a significant interaction ($F(2, 1056932) = 297.3, p < .0001$). Post-hoc Tukey HSD tests show that comments about men ($\mu = 0.305 \pm 0.190$) are still more dominant than comments about women ($p < .0001; d = 0.08$), but the effect size is still negligible. Comments on alt-right communities ($\mu = 0.309 \pm 0.189$) are significantly more dominant than those on the left ($\mu = 0.274 \pm 0.183, p < .0001, d = 0.19$) and right ($\mu = 0.301 \pm 0.189, p < .0001, d = 0.04$). Right-leaning comments remain more positive than those on the left ($p < .0001, d = 0.15$). While many interaction differences were significant, all differences in comment valence were negligible ($d < 0.2$), similarly to what was reported in the Trump-containing dataset.

When it comes to the cross-partisan comparison, again a three-way loglinear analysis of the sentiment output finds a final model retaining all effects with a likelihood ratio of $\chi^2(0) = 0, p = 1$, which again indicates the highest-order interaction is significant ($\chi^2(7) = 7140.6, p < .0001$). Chi-square tests on the two-way interactions within partisan groups finds that now only the alt-right subreddit has a significant association between politician gender and comment sentiment ($\chi^2(1) = 695.79, p < .0001, V = 0.03$), though, again, the strength of association is negligible; Odds ratio tests show that, in alt-right subreddits, men are now 1.18 times more likely to be described in positive sentiment than women ($95\% CI: 1.17 - 1.20, p < .0001$). There is again a significant association between partisanship and comment sentiment for female politicians

$(\chi^2(1) = 882.06, p < .0001, V = 0.06)$ and male politicians $(\chi^2(1) = 2648.4, p < .0001, V = 0.05)$.
